# Biotherapeutic potential of different fractions of cell-free supernatants from *Lacticaseibacillus rhamnosus* against *Pseudomonas aeruginosa*

**DOI:** 10.3389/fcimb.2025.1608897

**Published:** 2025-06-30

**Authors:** Marta Bianchi, Esingül Kaya, Viviana Logiudice, Giuseppantonio Maisetta, Aaron Curtis, Kevin Kavanagh, Giovanna Batoni, Semih Esin

**Affiliations:** ^1^ Department of Translational Research and New Technologies in Medicine and Surgery, University of Pisa, Pisa, Italy; ^2^ Department of Biology, Maynooth University, Maynooth, Co. Kildare, Ireland

**Keywords:** probiotics, *Pseudomonas aeruginosa*, biofilm, proteomics, immunomodulation, *Lacticaseibacillus rhamnosus*, PBMC

## Abstract

**Introduction:**

Due to their content of multiple antimicrobial bioactive substances, cell-free supernatants (CFS) from lactic acid bacteria are emerging as novel antimicrobials. We have previously demonstrated that CFS from *Lacticaseibacillus rhamnosus* exert strong antibacterial and antibiofilm activity against *Pseudomonas aeruginosa* isolated from chronic infections. Herein, we sought to identify the CFS fraction(s) responsible for such activities and characterize the same CFS in terms of immunomodulatory properties and protein content.

**Methods:**

A *P. aeruginosa* clinical isolate was used in the study. CFS fractions were obtained by 3 kDa cut-off size-exclusion filtration. The antibacterial and antibiofilm activity of unfractionated and fractionated CFS was tested on planktonic and biofilm-associated *P. aeruginosa* using colony-forming unit enumeration, crystal violet staining, and confocal microscopy. Label-free qualitative proteomic was performed using a shotgun approach with mass spectrometry to characterize the protein content of the CFS. Additionally, the immunomodulatory effects of the CFS were evaluated on human peripheral blood mononuclear cells (PBMC) stimulated with *P. aeruginosa* lipopolysaccharide (LPS) or biofilm-derived *P. aeruginosa* cells.

**Results:**

Both antibacterial and antibiofilm activities were mainly, but not exclusively, ascribed to the low molecular weight CFS fraction (≤ 3 kDa), which contained most of the lactic acid, suggesting a major role of this component in the antimicrobial effect of CFS. The > 3 kDa fraction alone was almost inactive but displayed a synergistic antibacterial effect when reconstituted with the ≤ 3 kDa fraction. Proteomics analysis of CFS revealed the presence of cell wall hydrolases, suggesting that these enzymes might contribute to the antibacterial activity observed in the reconstituted fractions. Following 6 h stimulation of PBMC with LPS or biofilm-derived *P. aeruginosa*, a marked anti-inflammatory effect was exhibited by unfractionated CFS as well as ≤ 3kDa fraction at non-toxic concentrations, while the > 3kDa fraction was found to induce the production of IL-6, TNF-α, and to a lesser extent of IL-10.

**Conclusion:**

The obtained results support that, due to their multiple antimicrobial and anti-inflammatory effects, probiotic metabolites might represent a promising strategy for the prevention and/or treatment of chronic infections with an intense inflammatory response such as those caused by *P. aeruginosa*.

## 1 Introduction

Recently, the World Health Organization (WHO) released its updated Bacterial Priority Pathogens List (BPPL 2024) (WHO Bacterial Priority Pathogens List, 2024). Although *Pseudomonas aeruginosa* was downgraded from the critical to the high-risk group, it remains one of the major human pathogens causing high morbidity and mortality, especially in immunocompromised individuals, hospitalized patients and those with cystic fibrosis (CF) (Reynolds and Kollef, 2021). The bacterium is involved in a wide variety of human infections that affect the lungs, skin, soft tissues, urinary tract and the bloodstream (Reynolds and Kollef, 2021). The therapy of *P. aeruginosa* infections is particularly challenging due to the intrinsic and acquired resistance of the bacterium to many antibiotic classes (Sathe et al., 2023). Strains sensitive to only colistin are frequent in many hospitals across the world and pan-drug resistance strains have also been reported (Ozma et al., 2022). Besides its resistance profile, *P. aeruginosa* produces a plethora of quorum sensing (QS) regulated virulence factors and exhibits a marked ability to form biofilms in infectious sites, adapting extraordinarily well to disparate host environments (Liao et al., 2022; Tuon et al., 2022). The *P. aeruginosa* biofilm not only is highly tolerant to antimicrobials, but it also induces an aberrant host inflammatory response that leads to intense tissue damage and loss of function. In this regard, it has been recently reported that during chronic lung infection of CF patients, *P. aeruginosa* undergoes changes in its lipopolysaccharide (i.e., endotoxin) that triggers increased pro-inflammatory cytokine production by human immune cells (Hofstaedter et al., 2024).

The significant difficulties in the treatment of *P. aeruginosa* infections and the few potential treatments in the development pipeline demand innovative approaches to address the impact of multi-drug-resistant strains of *P. aeruginosa* on health care (WHO Bacterial Priority Pathogens List, 2024). New treatment strategies under evaluation include inhibitors of QS, use of biofilm-degrading enzymes, interference with iron uptake, repurposed drugs, herbal medicine, or bacteriophages (Pires et al., 2015; Liao et al., 2022; Di Bonaventura et al., 2023; Sathe et al., 2023). The intense pro-inflammatory response evocated by *P. aeruginosa* suggests that new treatment strategies should not only target bacterial cells or reduce the virulence traits of the pathogen but possibly also exert an immunomodulatory effect.

An emerging innovative approach to combat *P. aeruginosa* infections is the use of probiotic bacteria or their bioactive products also referred to as postbiotics (Batoni et al., 2023a; Díaz et al., 2020; Soleymani et al., 2024). For instance, intra-tracheal administration of lactobacilli has been demonstrated to protect mice from *P. aeruginosa* pulmonary infection, and significantly decrease the secretion of interleukin (IL)-6 and tumour necrosis factor-α (TNF- α) in bronchoalveolar lavage samples (Fangous et al., 2018). In line with these findings, we have recently reported that *Lactobacillus* probiotic strains can adhere to human lung epithelial cells, although at different efficiency, and prevent host-cell adhesion of lung isolates of *P. aeruginosa* by a likely exclusion effect (Batoni et al., 2023b). Live lactobacilli have been also topically administered for therapeutic purposes on *P. aeruginosa*-infected wounds in both animal models and humans, with positive outcomes in terms of reduction of bacterial load and wound healing (Valdéz et al., 2005; Peral et al., 2009).

In order to overcome the possible risks associated with the administration of live probiotics, especially in immunocompromised individuals, over the last few years the concept that the viability of probiotics is not strictly necessary for their beneficial effects has gained interest (Piqué et al., 2019). Furthermore, it has become evident that byproducts released by probiotics through their metabolic activity are at least partially responsible for the health-promoting effects ascribed to probiotics, boosting investigations on their possible use as biotherapeutics (Nataraj et al., 2020; Żółkiewicz et al., 2020; Ozma et al., 2024b). In this regard, we have recently demonstrated that cell-free supernatants (CFS) from different strains of lactobacilli exert a strong and fast antibacterial activity towards lung isolates of *P. aeruginosa* and ability to reduce the expression/production of major *P. aeruginosa* virulence factors (Pompilio et al., 2024). Among them, CFS derived from *Lacticaseibacillus rhamnosus* GG were one of the most active and, when tested *in vivo* in the invertebrate *Galleria mellonella* infection model, disclosed lack of toxicity and ability to prevent *P. aeruginosa* infection (Pompilio et al., 2024). CFS from *L. rhamnosus* were also demonstrated to affect *P. aeruginosa* wound isolates in mono-species or dual-species biofilms in an *in vivo*-like wound infection model (Kaya et al., 2024). Importantly, in the same study, CFS failed to induce *P. aeruginosa* resistance after 15 passages at sub-inhibitory concentrations, unlike what was observed with ciprofloxacin, an antibiotic often used for the treatment of *P. aeruginosa* infections (Kaya et al., 2024).

*L. rhamnosus* GG is one of the most well-characterized probiotic strains with recognized immunomodulatory, antimicrobial and mucosa-adhesion properties (Capurso, 2019). Although its beneficial effects has been mostly demonstrated in the gastrointestinal tract, its therapeutical potential has recently highlighted in several extra-intestinal contexts including the respiratory tract. For instance, early administration of *L. rhamnosus* GG has demonstrated to improve the outcome following *P. aeruginosa*–induced pneumonia, likely through an immunomodulatory effect (Khailova et al., 2013). The targeted delivery of *L. rhamnosus* GG to the respiratory tract has been also recently investigated by developing a dry powder inhaler formulation of the probiotic intended to target *P. aeruginosa* infection in bronchiectasis maintenance therapy (Tran et al., 2024). The study established the feasibility of delivering the *L. rhamnosus* GG by the developed formulation without adversely affecting its anti-pseudomonal activities, further supporting the use of such probiotic as innovative strategy against *P. aeruginosa* infections.

Given this promising background, the aim of the present study was to further investigate the potential of *L. rhamnosus*-derived postbiotics in anti-infective therapy by identification of the bioactive fractions against planktonic and biofilm-associated *P. aeruginosa*. Furthermore, the immunomodulatory effect of both unfractionated and fractionated CFS towards human-peripheral blood mononuclear cells (PBMC) was investigated using as stimuli *P. aeruginosa* LPS or biofilm-derived *P. aeruginosa* cells. Overall, the results obtained demonstrated that both the antibacterial and the antibiofilm potential of the CFS are mainly ascribed to low molecular weight components (≤ 3 kDa) and are highly dependent on acidic conditions. The same low molecular weight components were also responsible for the anti-inflammatory activity at non-toxic concentrations, adding further evidence to postbiotics as novel treatment options in future.

## 2 Materials and methods

### 2.1 Bacterial strain and culture conditions

*P. aeruginosa* used throughout this study (PA1) is a non-mucoid clinical isolate, part of a collection of strains from the Microbiology laboratory of the University of Pisa. It was originally isolated from the sputum of a chronically infected cystic fibrosis patient (Pompilio et al., 2024). *L. rhamnosus* was isolated from a commercially available Italian product as previously described (Pompilio et al., 2024). Species identification for PA1 and *L. rhamnosus* was carried out using MALDI-TOF mass spectrometry (Bruker Daltonics, Bremen, Germany). The laboratory reference strain of *P. aeruginosa*, PAO1 (ATCC 15692) was also used to prepare ultraviolet-inactivated biofilm-derived bacteria. To prepare stock cultures, PA1 and PAO1 were cultivated on Tryptone Soy Agar (TSA, Oxoid, Basingstoke, Hampshire, UK), while *L. rhamnosus* on De Man–Rogosa–Sharpe agar (MRSA, Oxoid, Basingstoke, Hampshire, UK) and stored at −80°C until use using the Microbank™ system (Pro-Lab Diagnostics, Richmond Hill, ON, Canada) according to the manufacturer’s instructions.

### 2.2 Preparation of CFS

*L. rhamnosus* was grown in De Man–Rogosa–Sharpe broth (MRSB, Oxoid) under shaking conditions for 48 hours, at 37°C, in microaerophilic conditions. Cultures in stationary phase growth (OD_600_ approximately 6.5-7.0) were centrifuged at 4000 × g for 10 min, and the supernatants were filtered to sterilization using 0.22 μm filters (Euroclone SpA, Pero, Milan, Italy). The resulting CFS were aliquoted and stored at −20°C until needed.

### 2.3 Size-exclusion filtration of CFS

CFS were subjected to size-exclusion filtration to separate fractions based on molecular weight. Filtration was performed using Vivaspin^®^ 2 concentrators with a 3 kDa molecular weight cut-off polyethersulfone membrane (Sigma-Aldrich, St. Louis, MO, USA). Briefly, the CFS (3 ml) were loaded into the concentrator and centrifuged at 4000 × g for 30 min at room temperature, as per the manufacturer’s instructions. The filtrate (≤ 3 kDa) was collected, and the retentate (> 3 kDa) was obtained by reverse spin, centrifuging 3000 × g for 2 min. To adjust the volumes of the fractions to their original levels, MRSB was subjected to size-exclusion filtration in the same manner as above. The > 3 kDa fraction of MRSB was used to reconstitute the ≤ 3 kDa CFS fraction, while the ≤ 3 kDa MRSB fraction was used to reconstitute the > 3 kDa CFS fraction, so that each fraction reacquired the initial volume of 3 ml. Additionally, a recombined sample was prepared by mixing the ≤ 3 kDa and > 3 kDa CFS fractions to reach the original volume of the unfractionated sample. All CFS fractions were sterilized by passage through 0.22 μm filters, divided into aliquots and stored at −20°C until further use.

### 2.4 Evaluation of pH and lactic acid content

The pH of the whole CFS and their fractions was measured using the pH 7 Vio pH meter equipped with an XS Sensor Micro P S7 probe (Chimica Toscana, Italy). Measurements were performed on three independent batches of CFS.

Lactic acid concentrations in the CFS were determined using the L-Lactate Assay Kit (MAK329-1KT, Sigma-Aldrich, St. Louis, MO, USA), following the manufacturer’s instructions. This analysis was also conducted on three independent batches of CFS.

### 2.5 Activity of unfractionated and fractionated CFS against planktonic *P. aeruginosa*

PA1 in the exponential growth phase was adjusted to a final concentration of approximately 1×10^7^ CFU/mL in Tryptone Soy Broth (TSB) (Oxoid), based on the optical density at 600 nm. For the assay, bacterial suspensions in TSB were incubated for 30 min at 37°C in shaking conditions (Eppendorf ThermoStat™ C) with either unfractionated CFS, ≤ 3 kDa CFS fraction, > 3 kDa CFS fraction, or a recombined CFS sample. All conditions were tested at a ratio of 1:4. Control samples were represented by untreated bacteria incubated in the presence of MRSB diluted 1:4 with TSB. Following incubation, samples were serially diluted in phosphate-buffered saline (PBS, Euroclone SpA) and plated on TSA for colony-forming unit (CFU) enumeration.

### 2.6 Activity of unfractionated and fractionated CFS against biofilms of *P. aeruginosa* via crystal violet staining

The effects of unfractionated and fractionated CFS on PA1 mature biofilms were assessed using a crystal violet (CV) staining assay. An overnight culture of PA1 in TSB was diluted 1:100 in TSB and aliquots of 100 μl were added to wells of 96-well flat-bottom polystyrene microtiter plates (Euroclone SpA) and incubated for 24 h at 37°C in static conditions to allow biofilm formation. After incubation, non-adherent cells were gently removed by washing the wells twice with sterile PBS. Subsequently, 200 µL of unfractionated CFS, ≤ 3 kDa CFS fraction, > 3 kDa CFS fraction, or recombined CFS all diluted 1:4 and 1:8 in TSB were added to the wells and incubated for 24 hours at 37°C in static conditions. Wells with MRSB diluted 1:4 and 1:8 in TSB served as positive controls. Following treatment, wells were washed twice with PBS to remove residual CFS and non-adherent cells. Washed biofilms were fixed at 60°C for 1 hour. Biofilms were stained with 200 µL of 0.5% (w/v) crystal violet (bioMérieux, Florence, Italy) for 15 min at room temperature, empty wells were stained to be used as a negative control. Excess dye was removed by washing the wells with sterile water until no dye was seen in the washing water. The plate was dried at 37°C for 30 min. Biofilms were solubilized using 200 µL of 33% (v/v) acetic acid (Merck), and the absorbance was measured at 570 nm using a microplate reader (Multiskan FC, Thermo-Fisher Scientific, Monza, Italy).

### 2.7 Activity of unfractionated and fractionated CFS against biofilms of *P. aeruginosa* via confocal microscopy

Biofilms of PA1 were analyzed using the Zeiss LSM 900 Airyscan 2 confocal laser-scanning microscope (Carl Zeiss AG, Oberkochen, Germany) from the Center for Instrument sharing of the University of Pisa (CISUP, https://cisup.unipi.it/). In brief, 200 μl of PA1 stationary phase culture, diluted 1:100 in TSB was added to 8-well ibiTreat polymer multiwells (ibidi GmbH, Gräfelfing, Germany), and biofilms were grown for 24 hours. Following two washes with PBS to remove planktonic bacteria, 240 µL of unfractionated CFS, ≤ 3 kDa CFS fraction, or > 3 kDa CFS fraction, 1:4 and 1:8 in TSB were added to the wells and incubated for 24 hours at 37°C in static conditions. Wells with MRSB diluted 1:4 and 1:8 in TSB served as positive controls. After the incubation period, non-adherent cells were removed by carefully washing the biofilms with sterile PBS. The biofilms were then stained with Syto9, a green fluorescent dye for live cells, and propidium iodide, a red fluorescent dye that marks dead cells (Filmtracer™ LIVE/DEAD™ Biofilm Viability Kit, Thermo Fisher Scientific). The stained biofilms were visualized using confocal microscopy using a 63 × oil immersion objective, and images were captured to evaluate the viability and structure of the biofilms following treatment with unfractionated and fractionated CFS.

### 2.8 Preparation of ultraviolet-inactivated *P. aeruginosa* biofilm bacteria (UVBF)

Biofilms of the reference strain *P. aeruginosa* PAO1 were prepared in 24-well plates as described in section 2.6 with some modifications. Briefly, overnight bacterial cultures in TSB were diluted 1:100 in RPMI 1640 (Euroclone SpA), and 1 mL of the suspension was added to each well of 24 well plates. Following incubation at 37°C for 24 hours to allow biofilm formation, non-adherent cells were gently removed by washing the wells twice with sterile PBS. Biofilm-associated bacteria were disrupted by scraping with the tip of a pipette and resuspended in PBS. The suspension was then vortexed, sonicated, and vortexed again for 30 seconds to break up the biofilm structure. The resulting bacterial suspension was inactivated by exposure to ultraviolet (UV) light for 60 min, with gentle mixing after 30 min. The efficacy of inactivation was confirmed by plating aliquots on TSA and incubating at 37°C for 24 hours to verify the absence of bacterial growth. The UV-killed bacterial preparation was divided into aliquots and stored at -20°C.

### 2.9 PBMC isolation

Peripheral blood mononuclear cells (PBMCs) were isolated as previously described (Kaya et al., 2021) from the buffy coats of healthy donors following informed consent. The study was conducted in accordance with the Declaration of Helsinki, and the protocol was approved by the local Ethical Committee (Comitato Etico Area Vasta Nord-Ovest, CEAVNO, Protocol 34743, 28 June 2018). Blood was collected and diluted with PBS in a 1:1 ratio, containing 10% sodium citrate (v/v). The cell suspension was then layered on a density gradient solution (Lymphoprep, Cedarlane, ON, Canada) and centrifuged at 160 × g for 20 min at room temperature (RT). Following centrifugation, to eliminate platelets, the supernatant was gently removed without disturbing the mononuclear layer at the interface. The mononuclear layer was collected and subjected to a second centrifugation at 800 × g for 20 min. The PBMC were then washed two times with RPMI 1640 medium (Euroclone, Pero, Milan, Italy) to remove any remaining impurities and resuspended in complete RPMI medium (10% Autologous Human Plasma and 2 mM L-glutamine in RPMI 1640).

### 2.10 PBMC stimulation, cytokine profiling and cytotoxicity assessment

Isolated PBMCs were seeded at a density of 100,000 cells per well in complete RPMI medium. They were exposed to whole CFS, CFS ≤ 3 kDa and CFS > 3 kDa fractions, either alone or in combination with 5 μg/mL lipopolysaccharide (LPS) from *P. aeruginosa* (strain ATCC 27316, Merck) or with 1x10^6^ UVBF/well for 6 h. Following incubation, the plates were centrifuged, and the supernatants were carefully collected and stored at −20°C for subsequent cytokine quantification.

Cytokine levels (IL-6, TNF-α, IL-10) in the supernatants were measured using the LEGENDplex™ Multi-Analyte Flow Assay Kit (BioLegend, San Diego, CA, USA), which allows the simultaneous detection of multiple cytokines via flow cytometry, following the manufacturer’s protocol. Samples were analyzed using a BD Accuri C6 flow cytometer (BD Biosciences, Milan, Italy). The data were evaluated with LegendPlex v8.0 software (BioLegend Inc.), and cytokine concentrations were determined from a standard curve. The sensitivity of each cytokine tested was as follows: IL-6 1.1 + 1.0 pg/mL, TNF-α 0.9 + 0.6, IL-10 0.9 + 0.4.

To assess cytotoxicity, the pelleted cells from the same experiment were stained with 0.1 μg/mL propidium iodide (PI, Merck, Milan, Italy) at room temperature for 5 min. Flow cytometric acquisition was performed without gating, with 50,000 events recorded per sample using a BD Accuri C6 flow cytometer. The percentage of PI-positive cells was calculated using BD Accuri C6 software (version 1.0.264.21). Cell viability was determined with the formula:

Viability (%) = 100 − [100 × (%PI-positive sample − % negative control)(100 − % negative control)].

### 2.11 Protein extraction and purification from CFS

Protein extraction from CFS was performed as outlined previously (Margalit et al., 2022). To summarize, protein concentration in supernatants was initially estimated using the Bradford assay (Bio-Rad, Hercules, CA, USA). Proteins were precipitated by overnight acetone precipitation at a 1:6 ratio, then resuspended in 25μl of lysis buffer (8 M urea, 2 M thiourea, 0.1 M Tris-HCl, pH 8.0) and prepared with MilliQ water (MerckMillipore, Darmstadt, Germany). Protein quantification was conducted using the Qubit system (Invitrogen, Carlsbad, CA, USA) in accordance with the manufacturer’s instructions. Proteins were reduced with 1 μl of 0.5 M DTT (Sigma-Aldrich, St. Louis, MO, USA) at 56°C for 20 min, followed by alkylation with 2.7 μl of 0.55 M iodoacetamide (Sigma-Aldrich) in the dark at room temperature for 15 min. Digestion was performed with 1μl Protease Max Surfactant Trypsin Enhancer (1% w/v, Promega, Madison, WI, USA) and 1μl Sequence Grade Trypsin (Promega) at 37°C for 18 hours. Digestion was terminated with 100% TFA (Thermo Fisher Scientific, Waltham, MA, USA), and peptides were purified using C18 Spin Columns (Pierce, Thermo Fisher Scientific) following the manufacturer’s protocol. The purified peptides were dried in a SpeedVac concentrator (Thermo Scientific Savant DNA120) until mass spectrometry analysis.

### 2.12 Label-free qualitative proteomics analysis of CFS

Lyophilized proteins from CFS were resuspended to give a final concentration of 375 ng/ul of which 2 μl was loaded onto the Q-extractive mass spectrometer (Thermo Fisher Scientific, Waltham, MA, USA) using a 133 min reverse-phase gradient. Qualitative proteomic analysis of the CFS was performed using Proteome Discoverer software (version 1.4) and the Sequest HT algorithm (Thermo Scientific). Proteins identified were matched against the *L. rhamnosus* proteome downloaded from UniProtKB (identifier 47715, protein count 2,732, date 05/11/2024, https://www.uniprot.org/taxonomy/47715) obtained from UniProt (The UniProt Consortium et al., 2025). To verify the specificity of the analysis, a control sample, represented by MRSB, was processed in the same way as CFS. As expected, the Proteome Discoverer software did not identify any proteins in the control sample. Only protein identifications with high confidence were included, peptides with fewer than 2 matching unique peptides were excluded from the analysis to ensure result accuracy and reliability.

### 2.13 Statistical analysis

Data were analyzed using GraphPad Prism 9 (Dotmatics, Boston, MA, USA). All experiments, including evaluations of planktonic *P. aeruginosa* growth, biofilm activity, pH, and lactic acid content, cytokine analysis and cytotoxicity were performed at least three times independently. Differences between mean values were assessed using One-way analysis of variance (ANOVA), followed by Tukey-Kramer *post hoc* tests for parametric multiple comparisons or with Kruskal-Wallis non-parametric test with Dunn’s multiple comparisons. A p-value of < 0.05 was considered statistically significant.

## 3 Results

### 3.1 Antibacterial activity of unfractionated and fractionated CFS towards planktonic *P. aeruginosa*

**Figure 1:**
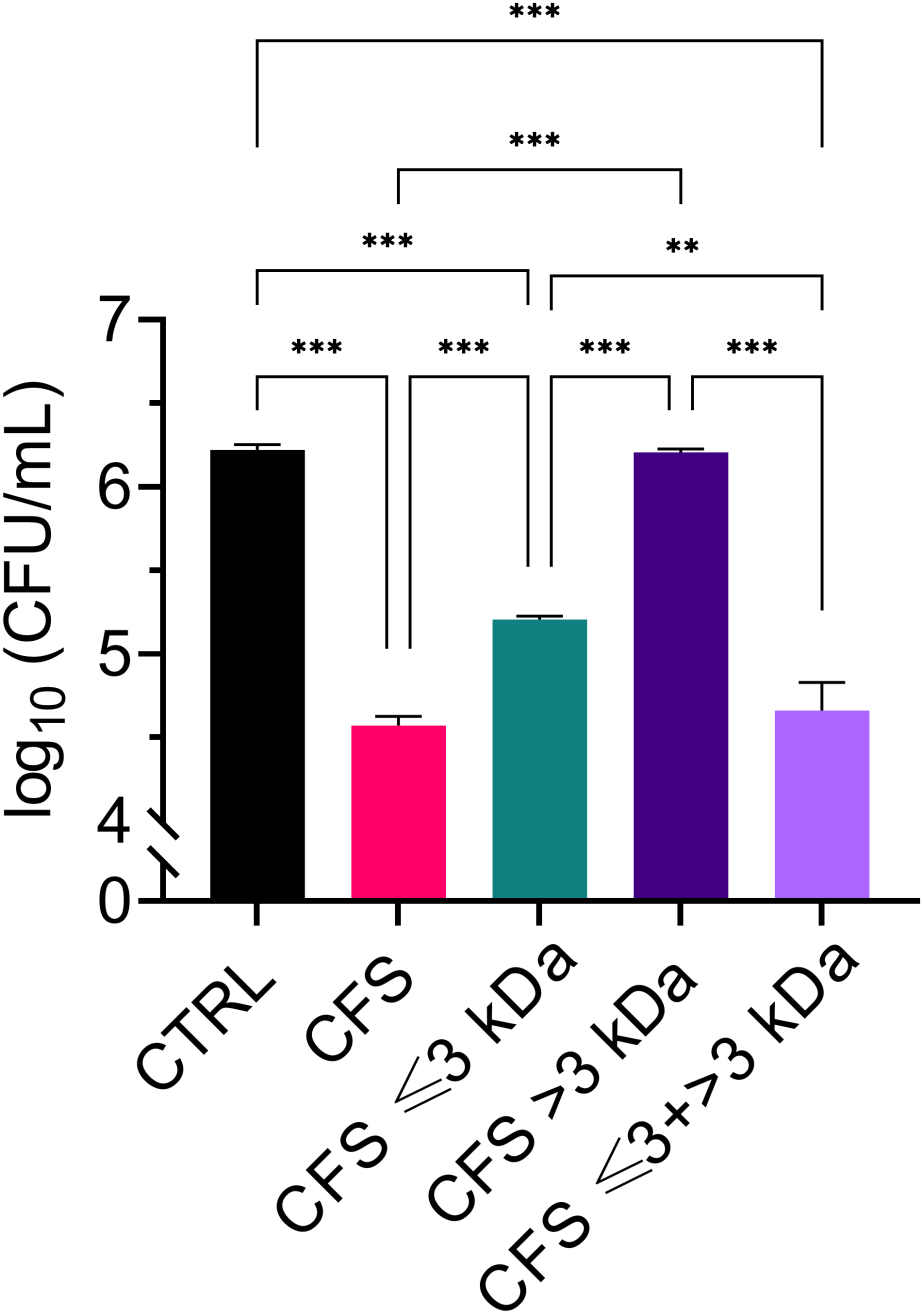
Killing ability of CFS, CFS ≤ 3 kDa, CFS > 3kDa and CFS ≤ 3 kDa + > 3 kDa at dilutions 1:4 in TSB against planktonic *P. aeruginosa*, strain PA1. The viable count was evaluated by measuring CFU/mL of PA1 following 30 min of incubation with the treatments. CTRL represents untreated bacteria incubated in the presence of TSB with 25% MRSB. Data are reported as mean ± standard error of the mean of three independent experiments. ∗∗p < 0.01; ∗∗∗p < 0.001 (One-way ANOVA followed by Tukey-Kramer *post hoc* test).

Cell-free supernatants from *L. rhamnosus* (CFS) were subjected to size fractionation via ultrafiltration on membranes with a molecular weight cut-off of 3 kDa. The two fractions obtained were referred to as CFS ≤ 3 kDa, and CFS > 3 kDa, respectively. A third fraction was obtained by combining CFS ≤ 3 KDa, and CFS > 3 kDa to obtain whole CFS, as a control for loss of activity during the fractionation process (CFS ≤ 3 + > 3 kDa). Antibacterial activity of unfractionated and fractionated CFS was tested towards a non-mucoid clinical isolate of *P. aeruginosa* originally isolated from the sputum of a chronically infected cystic fibrosis patient (Pompilio et al., 2024). In agreement with our previous studies (Pompilio et al., 2024), unfractionated CFS exerted a strong antibacterial activity, causing approximately 1.5 log CFU reduction in 30 min of incubation at 37°C (**Figure 1**). After fractionation, most but not all of the antibacterial activity was found in the low molecular weight fraction (CFS ≤ 3 kDa), while the high molecular weight fraction (CFS > 3 kDa) was found to be inactive. As shown in **Figure 1**, CFS ≤ 3 + > 3 kDa exerted an antibacterial activity comparable to unfractionated CFS.

### 3.2 Lactic acid content and pH of unfractionated and fractionated CFS

**Figure 2:**
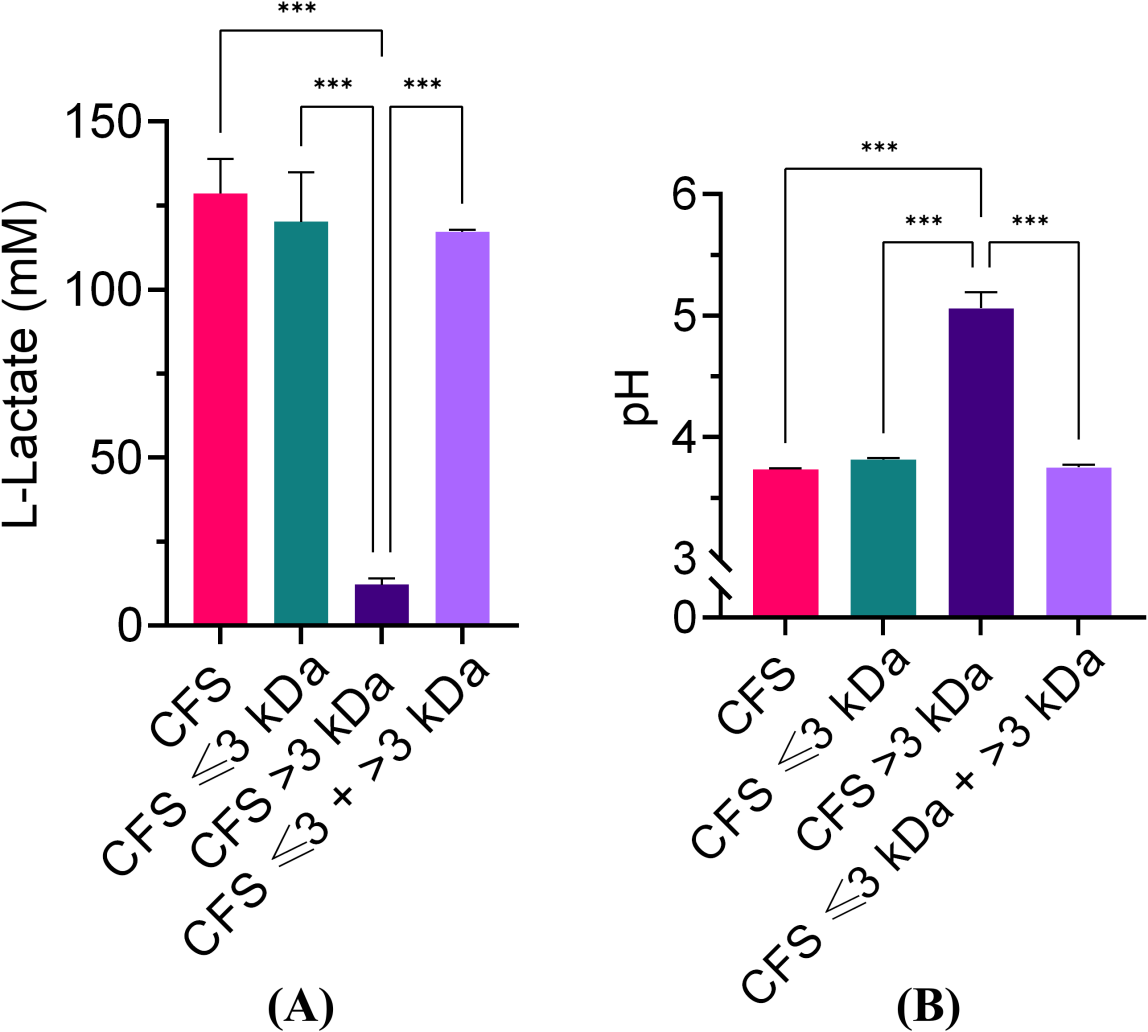
Lactic acid quantification and pH values of unfractionated and fractionated CFS. **(A)** Lactic acid quantification; **(B)** pH values. Data were obtained on three different preparations of CFS and related fractions and are reported as mean ± standard error of the mean. ∗∗∗p < 0.001 (One-way ANOVA followed by Tukey-Kramer *post hoc* test).

It is widely agreed that at least part of the antibacterial activity of lactobacilli is due to their ability to produce lactic acid and lower the pH of the culture (Mani-López et al., 2022). In agreement with this view, we have previously reported that adjusting the pH of CFS to 6.0 with NaOH results in a loss of antibacterial activity against *P. aeruginosa* (Pompilio et al., 2024). To gain further insights into the role of lactic acid in the antibacterial activity of CFS, herein we proceeded to evaluate the lactic acid content and the pH value of unfractionated and fractionated CFS. Un-fractionated CFS, CFS ≤ 3kDa, and CFS ≤ 3kDa + > 3kDa had comparable and elevated lactic acid content (exceeding 100 mM), while the amount of lactic acid in CFS > 3kDa fraction was very low (**Figure 2A**). The results obtained inversely correlated with the pH value (**Figure 2B**) and the observed antibacterial activity (paragraph 3.1), suggesting that lactic acid plays a major role in the antibacterial effect of CFS.

**Figure 3:**
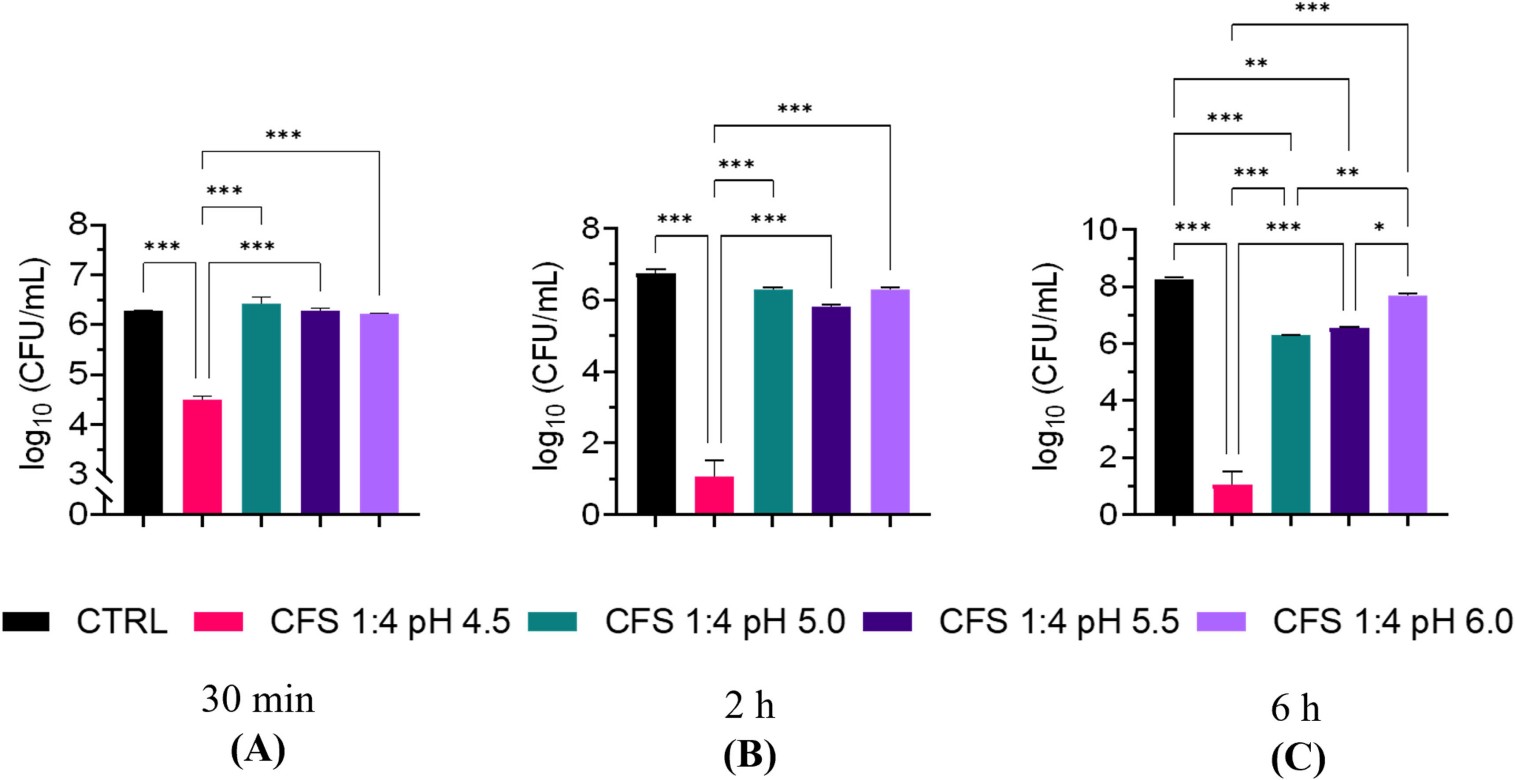
Killing ability of unfractionated CFS against *P. aeruginosa* at different pH and exposure times. **(A)** 30** min** exposure; **(B)** 2** h** exposure; **(C)** 6 h exposure. CFS were adjusted at different pH with NaOH and incubated with exponentially growing *P. aeruginosa* for different time intervals. Aliquots of the suspensions were serially diluted and plated on TSA for CFU enumeration. CTRL represents bacteria exposed to MRSB diluted 1:4 in TSB. Data are reported as mean ± standard error of the mean of two independent experiments in duplicates. ∗∗∗p < 0.001; ∗∗p < 0.01; ∗p < 0.05; (One-way ANOVA followed by Tukey-Kramer *post hoc* test).

The effect of pH on the antimicrobial potency of CFS was further investigated by exposing *P. aeruginosa* for 30 min, 2 h, and 6 h, to CFS whose pH had been adjusted with NaOH to pH 4.5, 5.0, 5.5 and 6.0, respectively. As shown in **Figure 3**, at all three time points, CFS at pH 6.0 almost completely lost its killing capacity. A marked and time-dependent reduction in the CFU number was observed with CFS at pH 4.5 (**Figures 3A–C**). At pH 5.0 and 5.5, *P. aeruginosa* viable counts were similar to the untreated control, after 30 min and 2 h of exposure (**Figures 3A, B**), while at 6 h of exposure, a statistically significant reduction in the CFU number was observed at both these pH as compared to the untreated control (**Figure 3C**). Thus, increasing the incubation time allows CFS at pH 5.0 and 5.5 to exert a detectable antimicrobial effect (approximately 2 logs).

### 3.3 Evaluation of protein content of unfractionated CFS by label-free qualitative proteomics analysis

**Figure 4:**
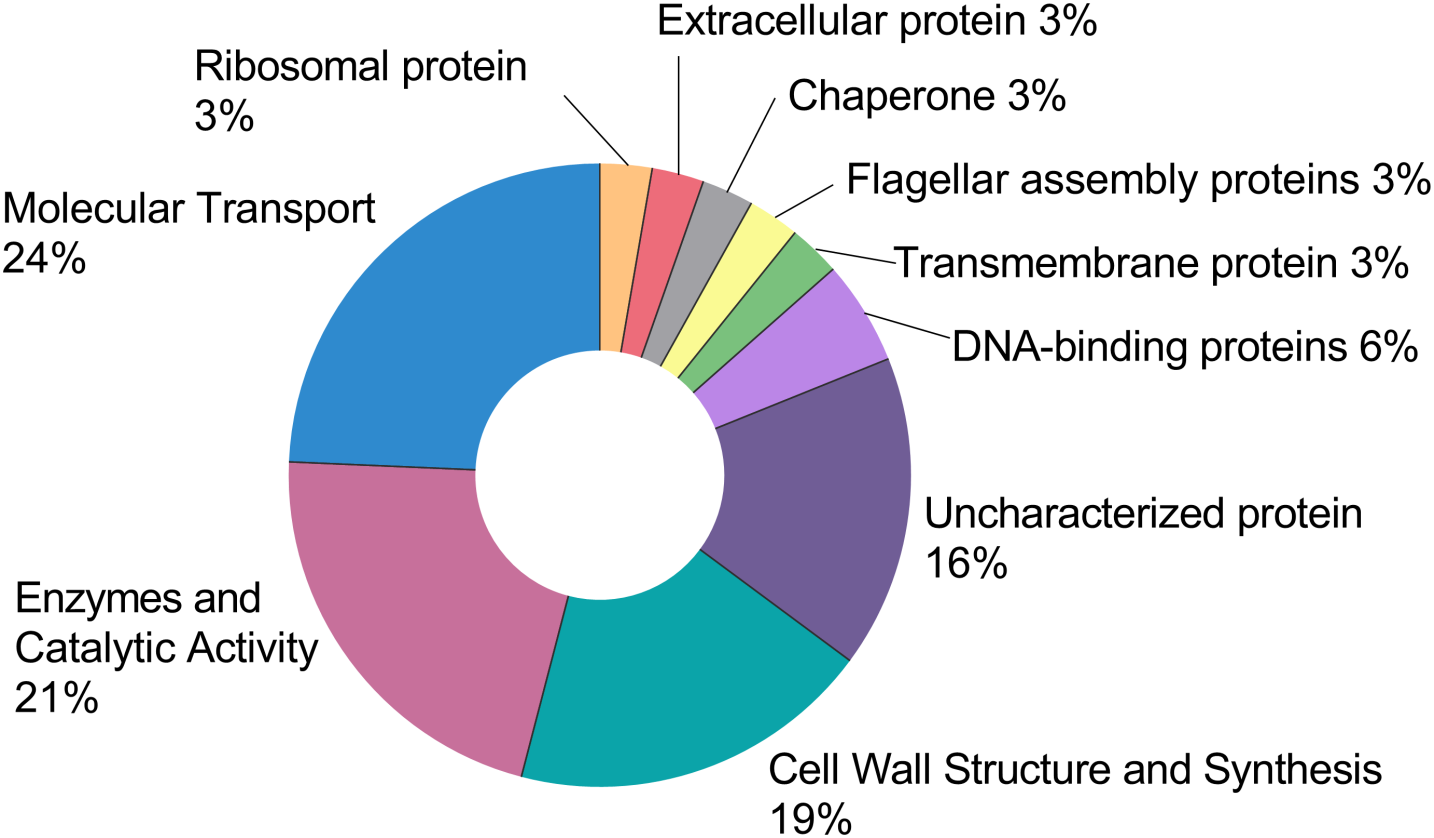
This pie chart represents the functional distribution of the 37 high-confidence proteins retrieved from the CFS. The full list can be found in the **Supplementary Data**. Each protein was assigned a function and expressed as a percentage of the total.

Using label-free qualitative proteomic analysis a total of 37 high-confidence proteins were identified from the CFS. The functional distribution of these proteins is summarised in **Figure 4**, and the complete list is provided in **Supplementary Table 1**. Among the identified proteins, 24% (9 proteins) were found to be transport-related, including eight proteins of the ATP-Binding Cassette (ABC) transporter family and one MMPL family transporter. Additionally, 21% (8 proteins) were identified as enzymes, which include 6 cell wall hydrolases, a serine protease, and N-acetylmuramoyl-L-alanine amidase. Nineteen percent (7 proteins) were involved in cell wall structure and synthesis, including three LytR-CpsA-Psr (LCP) family proteins, two penicillin-binding proteins (PBPs), a transpeptidase, and a serine-type D-Ala-D-Ala carboxypeptidase. Sixteen percent (6 proteins) were uncharacterized, with no currently assigned function. Furthermore, 5% (2 proteins) are DNA-binding proteins, specifically DNA-binding protein HU and a YxeA family protein. Lastly, other proteins identified include a transmembrane protein of the LemA family protein, a flagellar assembly protein, a chaperone protein, an extracellular protein, and a ribosomal protein.

### 3.4 Ability of unfractionated and fractionated CFS to reduce preformed biofilms of *P. aeruginosa*

**Figure 5:**
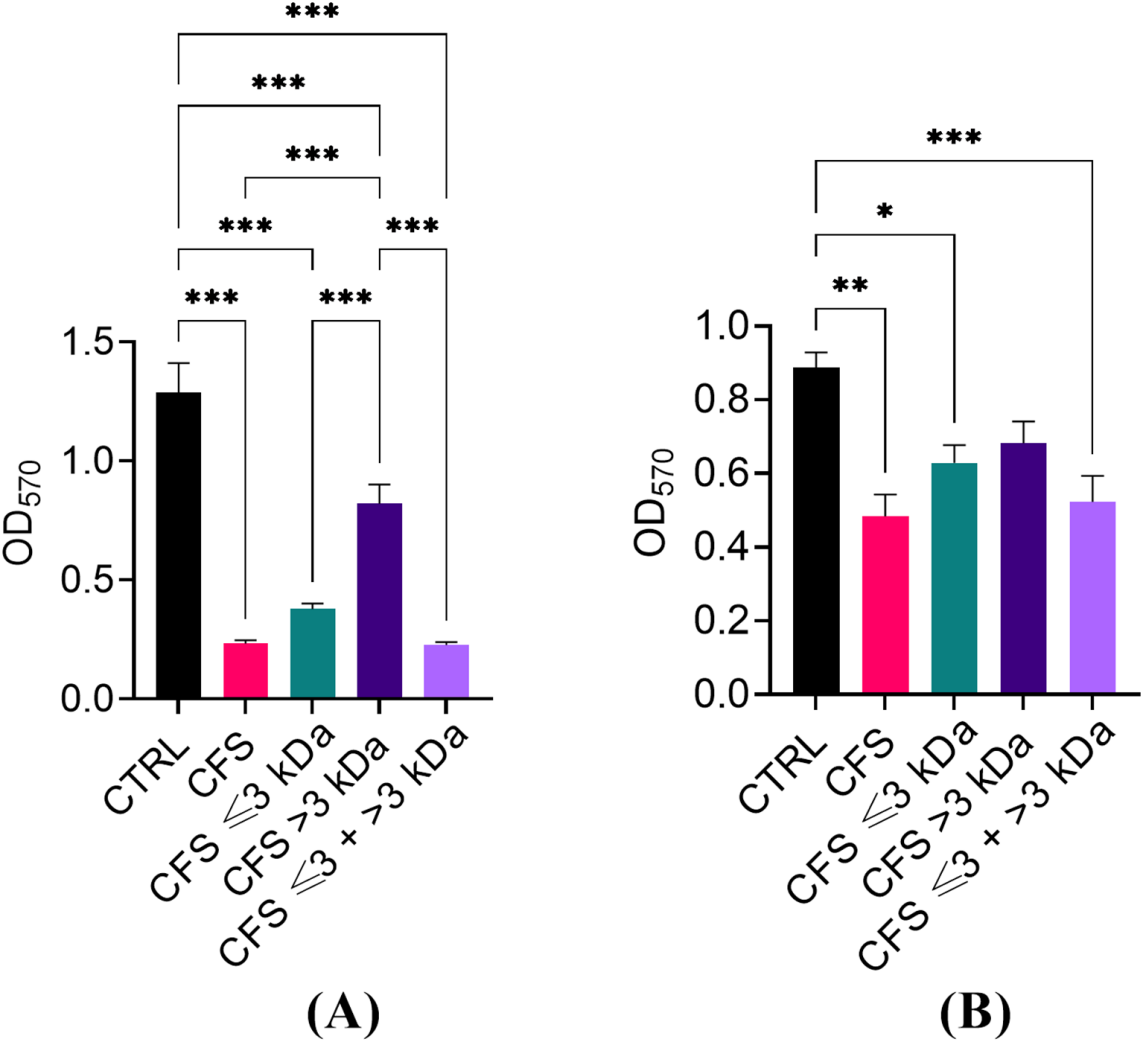
Ability of CFS, CFS ≤ 3kDa, CFS > 3kDa and CFS < 3kDa + > 3kDa to reduce the biomass of 24 h-old biofilms of *P. aeruginosa*, strain PA1. **(A)** Dilution 1:4 in TSB; **(B)** dilution 1:8 in TSB. The OD570 was measured following biofilm staining with crystal violet. CTRL represents untreated biofilms incubated in the presence of 25% **(A)** or 12.5% **(B)** of MRSB in TSB. Data are reported as mean ± standard error of the mean of three independent experiments. *p < 0.05; **p < 0.01; ***p < 0.001 (One-way ANOVA followed by Tukey-Kramer *post hoc* test).

The ability of unfractionated and fractionated CFS to reduce the biomass of preformed biofilms of *P. aeruginosa* was evaluated by a standard crystal violet assay. As shown in **Figure 5**, similarly to what was observed against planktonic *P. aeruginosa*, CFS ≤ 3 kDa fraction was the one retaining most of the antibiofilm activity, both when diluted 1:4 (**Figure 1A**) and 1:8 (**Figure 1B**). When tested at the dilution 1:4, even CFS > 3 kDa was able to reduce the biofilm biomass in a statistically significant manner as compared to CFS and CFS ≤ 3 kDa, following 24 h exposure.

**Figure 6:**
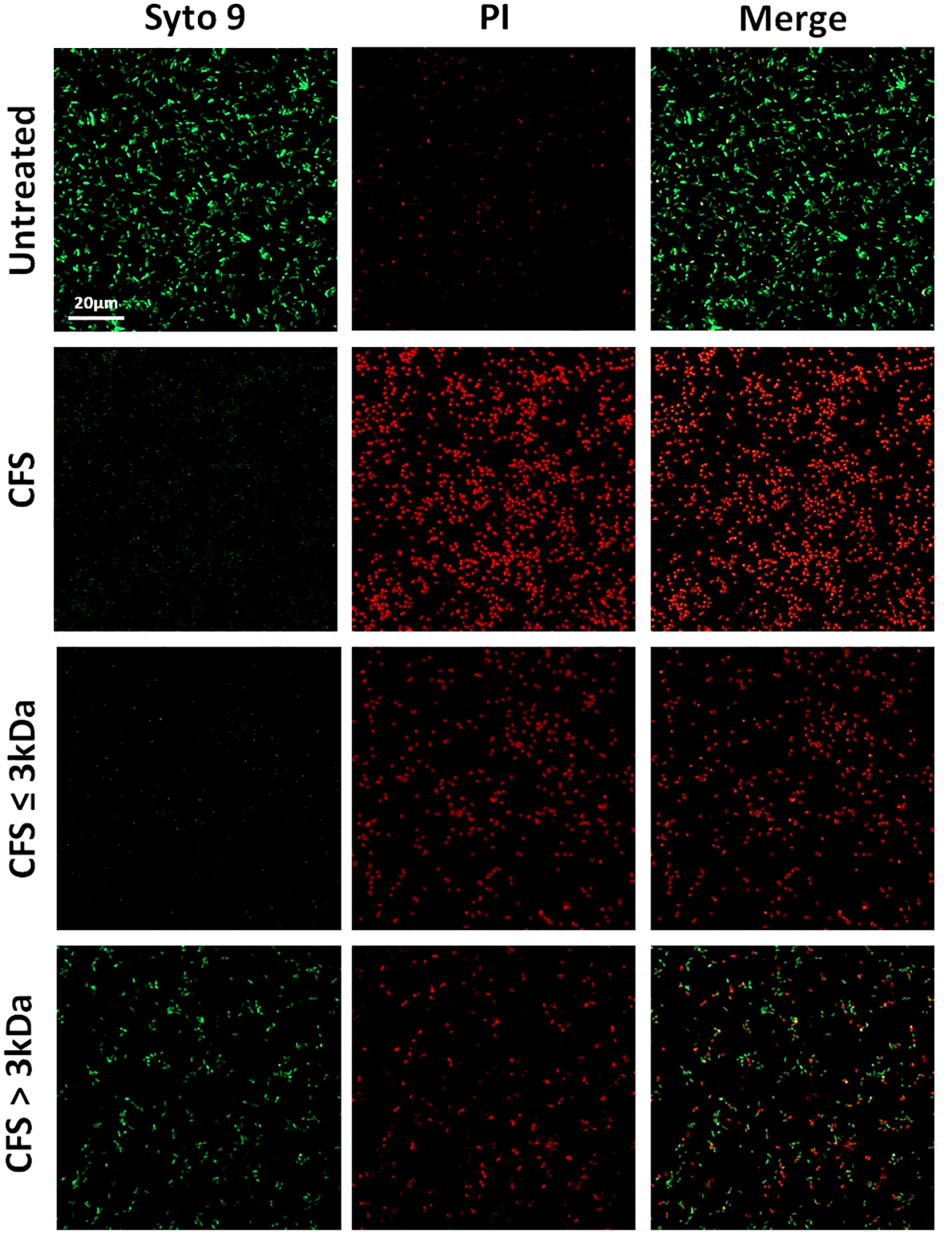
CLSM images of PA1 24h old biofilms treated with CFS or left untreated. The viability of PA1 biofilms were evaluated upon 24h treatment with unfractionated 1:4 diluted CFS, CFS ≤ 3kDa, and CFS > 3kDa or left untreated. After a wash to eliminate planktonic bacteria, PA1 biofilms were stained with green fluorescent labelled Syto 9 (488/500–540 nm) for live bacteria and with red fluorescent propidium iodide (PI, 488/600–650 nm) for dead bacteria. The figure shows results from a representative experiment. Scale bar = 20μm.

The antibiofilm activity of the fractions used at the dilution of 1:4 (**Figure 6**) was further evaluated by confocal microscopy through live/dead staining of untreated and treated biofilms. The results obtained confirmed a marked killing effect exerted by unfractionated CFS and CFS ≤ 3 KDa fraction and a milder, but still detectable, killing effect exerted by CFS > 3 KDa (**Figure 6**).

### 3.5 Ability of unfractionated and fractionated CFS to dampen the LPS-induced pro-inflammatory response

**Figure 7:**
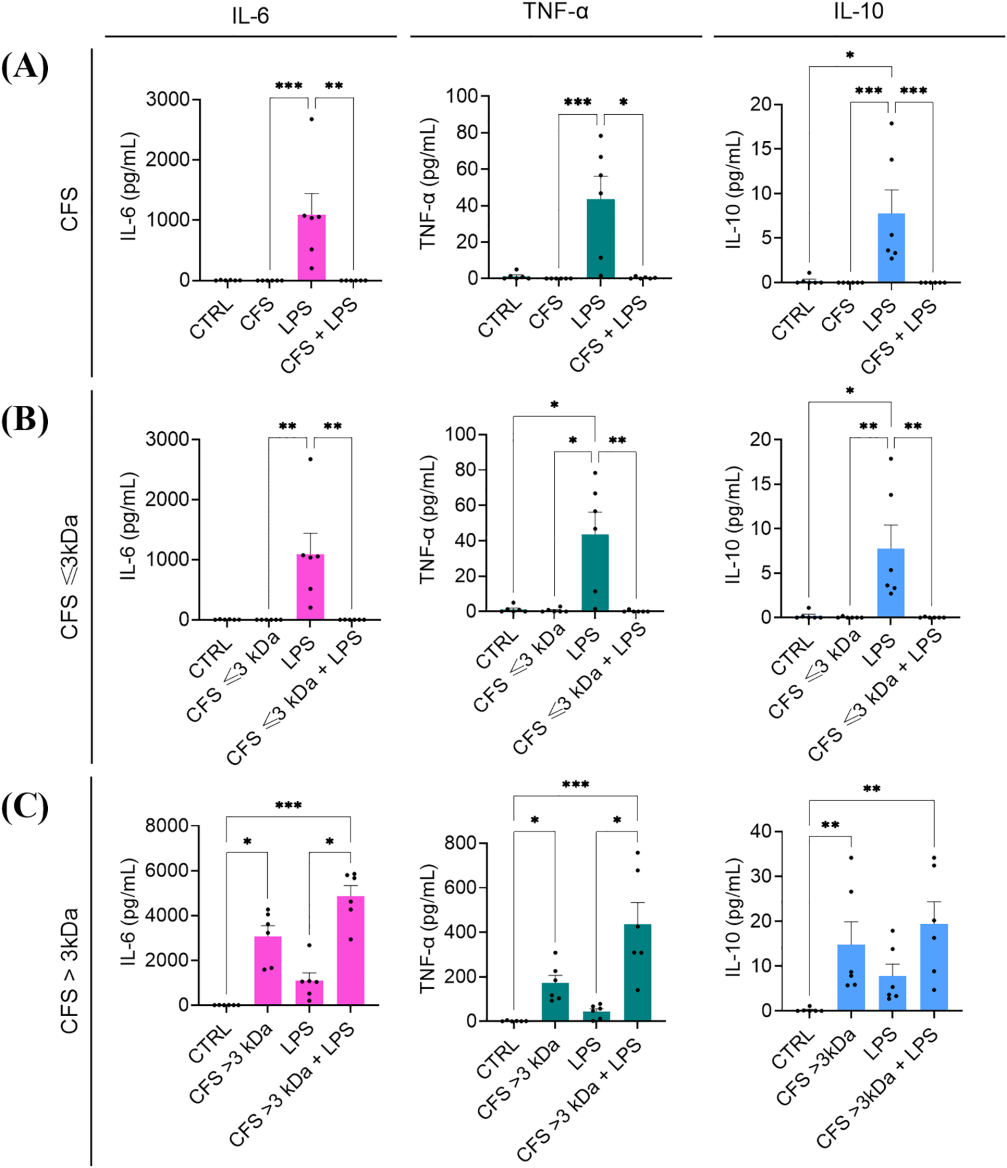
Immunomodulatory activity of CFS **(A)**, CFS ≤ 3kDa **(B)**, CFS > 3kDa **(C)** against human PBMC stimulated with *P. aeruginosa* LPS. All the CFS preparations, used alone or combined with LPS (5 μg/mL), were diluted 1:8 in complete RPMI and used as stimuli. After 6 h incubation at 37°C in humidified atmosphere at 5% CO2, culture supernatants were collected, and the content of IL-6, TNF-α and IL-10 was evaluated via flow cytometer-based multibead capture assay. CTRL represents unstimulated cells incubated in the presence of complete RPMI with 12.5% MRSB. Data represent mean ± standard error of the mean of three independent experiments. *p < 0.05; ∗∗p < 0.01; ∗∗∗p < 0.001 (Kurskal-Wallis followed by Dunn’s *post hoc* test).

The immunomodulatory effect of unfractionated and fractionated CFS was assessed against human PBMC stimulated for 6 h with *P. aeruginosa* LPS. As expected, when used alone LPS at the concentration of 5 μg/mL induced the production of IL-6 and TNF-α, and at a lower extent of IL-10 (**Figures 7A–C**). Co-incubation of LPS with both unfractionated CFS (**Figure 7A**) and CFS ≤ 3 kDa (**Figure 7B**) completely abrogated the release of cytokines in the culture supernatants. Neither CFS nor CFS ≤ 3 kDa, when used alone, stimulated a cytokine response (**Figures 7A, B**). In contrast, CFS > 3 kDa alone was able to induce the production of all three cytokines tested, although to a different extent, and this stimulatory capacity summed up to that of LPS (**Figure 7C** **).**

### 3.6 Ability of unfractionated and fractionated CFS to dampen the pro-inflammatory response of PBMC stimulated with biofilm-derived *P. aeruginosa*

**Figure 8:**
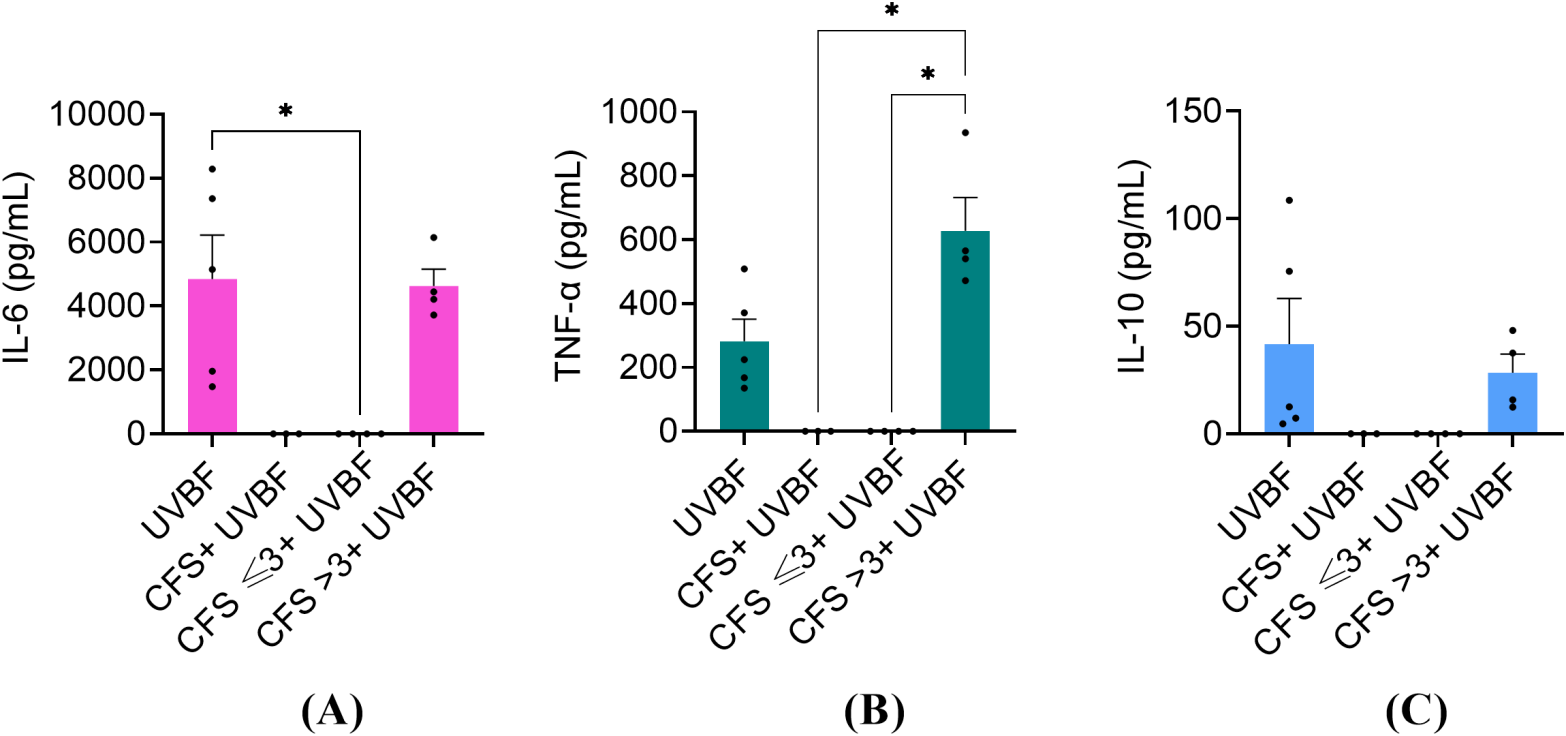
Immunomodulatory activity of CFS, CFS ≤ 3kDa, CFS > 3kDa against human PBMC stimulated with 1x10^6^ biofilm-derived *P. aeruginosa* (UVBF). All the CFS preparations were diluted 1:8 in complete RPMI and used alone or combined with UVBF. After 6 h incubation at 37°C in humidified atmosphere at 5% CO_2_, culture supernatants were collected, and the content of IL-6 **(A)**, TNF-α **(B)** and IL-10 **(C)** was evaluated via flow cytometer-based multibead capture assay. CTRL represents unstimulated cells incubated in the presence of complete RPMI with 12.5% MRSB. Data represent mean ± standard error of the mean of three independent experiments. *p < 0.05 (One-way ANOVA followed by Tukey-Kramer *post hoc* test).

Others and we (Ciornei et al., 2010; Kaya et al., 2021) have demonstrated that biofilm-embedded *P. aeruginosa* is particularly prone to induce a pro-inflammatory response. Therefore, we proceeded to investigate the ability of unfractionated and fractionated CFS to dampen the pro-inflammatory response of PBMC stimulated with biofilm-derived *P. aeruginosa*. To this aim, biofilm-derived bacteria, referred to as UVBF, were obtained by dislodging mature biofilms (24 h-old) of *P. aeruginosa*, killed by exposure to UV-light, and used as a stimulus for PBMC in the presence or not of CFS fractions. As shown in **Figure 8**, UVBF induced a strong pro-inflammatory response with mean levels of IL-6 and TNF-α in the culture supernatants exceeding 4000 pg/mL and 200 pg/mL, respectively. Similarly to what was observed with LPS, unfractionated CFS and CFS ≤ 3 kDa completely abrogated the cytokine response, while CFS > 3 kDa did not.

### 3.7 Cytotoxic potential of unfractionated and fractionated CFS against human PBMC

**Figure 9:**
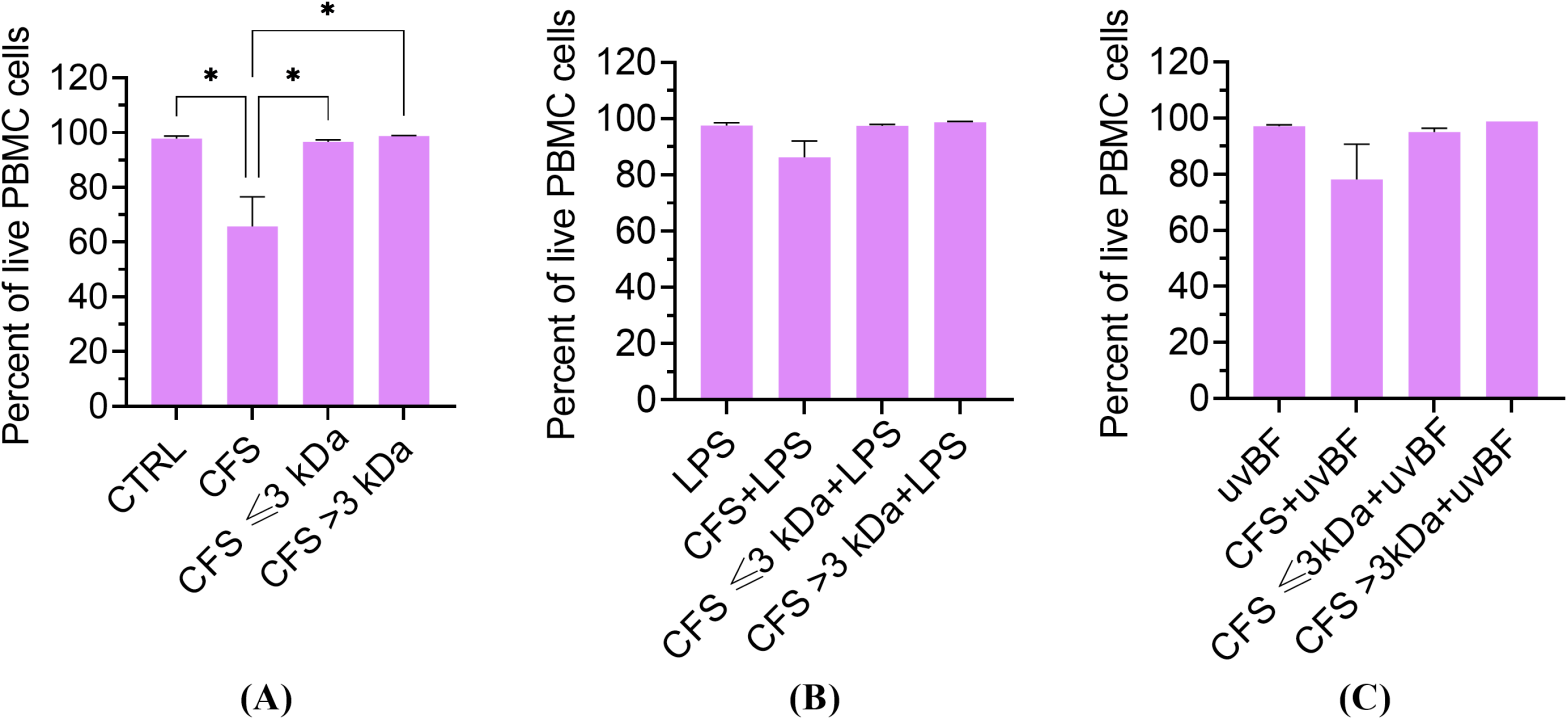
Cytotoxic potential of unfractionated and fractionated CFS used alone **(A)** or in combination with LPS **(B)** or UVBF **(C)** against human PBMC. PBMC were incubated for 6 h with the various preparations, all diluted at 1:8 in complete RPMI medium. Following incubation, cells were stained for 5 min with 0.1 μg/mL propidium iodide before evaluation of PI uptake by flow cytometry. CTRL represents untreated cells in MRSB diluted 1:8 in complete RPMI medium. Results are shown as mean ± standard error of the mean values (n=3). *p<0.05. (One-way ANOVA followed by the Tukey-Kramer *post hoc* test).

In order to verify that the strong anti-inflammatory effect exerted by the fractions was not due to a toxic effect against PBMC, all the fractions, used alone or combined with LPS or UVBF were tested in a cytotoxicity assay by measuring cell death by propidium iodide uptake and flow cytometry analysis (Crowley et al., 2016). As shown in **Figure 9**, none of the fractions or their combinations with LPS or UVBF exerted a cytotoxic effect against PBMC, except CFS used alone, which caused a 34 ± 11% loss of vitality.

## 4 Discussion

*P. aeruginosa* is one of the six bacterial pathogens of the ESKAPE group (*Enterococcus faecium*, *Staphylococcus aureus*, *Klebsiella pneumoniae*, *Acinetobacter baumannii*, *Pseudomonas aeruginosa*, and *Enterobacter* spp.) commonly associated with multi-drug resistance and mostly involved in nosocomial infections with high risk of mortality (Mulani et al., 2019). *P. aeruginosa* is also included by the WHO in the group of high priority pathogens for which the need for new antimicrobial strategies is urgently required (WHO Bacterial Priority Pathogens List, 2024). In the field of non-conventional antimicrobials, the use of postbiotics derived from health-promoting bacteria (i.e. probiotics) is recently attracting considerable interest (Yang et al., 2021; El Far et al., 2023; Do et al., 2025; Ozma et al., 2024a; Sornsenee et al., 2024). Currently, postbiotics are defined as intact non-viable microbes or cell fragments (cell surface proteins, polysaccharides, peptidoglycan), with or without metabolites (organic acids, enzymes, proteins, peptides, lipids) that provide a health benefit to the host (Vinderola et al., 2022). Yet, a global consensus on postbiotic definition is still lacking as the term postbiotics has rather long been referred to metabolites produced by probiotic bacteria (Aguilar-Toalá et al., 2021). Postbiotics are currently investigated for a wide range of clinical and commercial applications (Heniedy et al., 2024; Kumar et al., 2024) as well as functional bioactive compounds in food preservation and security (Mishra et al., 2024). Despite their potential in microbial control strategies, the mechanisms implicated are not fully elucidated and further research is needed to entirely explore their therapeutic potential. Therefore, in the present study, we aimed to investigate the biotherapeutic prospects of postbiotics from *L. rhamnosus* in the form of CFS against *P. aeruginosa*, by evaluating the antibacterial, antibiofilm and immunomodulatory properties of unfractionated and fractionated CFS. To further characterize CFS content, a detailed analysis of their protein content was also performed by label-free proteomic analysis.

In agreement with our previous reports (Kaya et al., 2024; Pompilio et al., 2024) unfractionated CFS exhibited a strong and fast antibacterial activity against planktonic *P. aeruginosa*. In addition, herein we demonstrated that such activity was mainly, attributable to the low molecular weight fraction (CFS ≤ 3kDa) although such fraction did not reach the killing levels of unfractionated CFS (CFU number of samples treated with CFS *versus* those treated with CFS <3kDa, p<0.001). On the other hand, the high molecular weight fraction (CFS > 3kDa) did not show any detectable antimicrobial activity, yet, when it was reconstituted with CFS ≤ 3kDa fraction killing levels comparable to those of unfractionated CFS were observed. These findings suggest that low molecular components of CFS (e.g. organic acids) are those mainly responsible for the killing effects, and that some of the components with molecular weight > 3 kDa may interact synergistically with low molecular weight components to fully exhibit their antimicrobial potential. One of the main metabolites produced by all lactic acid bacteria is lactic acid (molecular weight 0.09008 kDa). The CFS ≤ 3kDa fraction was the one with the highest content of lactic acid, and the lowest pH, while the CFS > 3kDa fraction had a lactic acid content close to the limit of detection and the highest pH. Thus, lactic acid contents and pH of the fractions correlated with the extent of their antibacterial activity, suggesting that both lactic acid and pH play a major role in the killing ability of CFS. Not only the pH, but also the exposure time seemed to play a role. Indeed, at early exposure time (30 min, 2h) only CFS with a net acidic pH (4.5) were able to exert a strong killing effect, while at longer exposure time (6 h) also CFS whose pH was adjusted to pH 5 or 5.5 reduced the viable bacterial count in a statistically significant manner as compared to the control. At pH 6.0 almost no antibacterial activity was detected against *P. aeruginosa* irrespective of the exposure time. It is reported that the undissociated form of lactic acid produced by lactobacilli can penetrate bacterial cell membranes by virtue of its lipophilic nature. Once in the neutral environment of the cytoplasm lactic acid dissociates and releases hydrogen ions that, in turn, lower the intracellular pH hindering vital bacterial cell functions and leading to cell death (Denkova et al., 2017; El Far et al., 2023). Interestingly it has been reported that lactic acid in addition to its antimicrobial property due to the lowering of the pH, also functions as a permeabilizer of the Gram-negative bacterial outer membrane and therefore may act as a potentiator of the effects of other antimicrobial substances (Alakomi et al., 2000). Furthermore, at a net acidic pH (between 2 and 4) the antibacterial activity of bacteriocins or bacteriocins-like proteins produced by certain lactobacilli is greatly enhanced, while their activity decreases as the pH increases (Bendjeddou et al., 2012; Kumar et al., 2016). Altogether, these observations could explain the lack of an evident antibacterial activity of the mildly acidic and lactic acid devoid CFS > 3kDa fraction, and, at the same time, the ability of some of its components to contribute to the overall antibacterial activity of CFS when reconstituted with the highly acidic and lactic acid rich CFS ≤ 3kDa fraction. Ongoing studies from our group comparing the effects on *P. aeruginosa* of lactic acid, used as a positive control, and unfractioned CFS will help to better discriminate the specific contribution of lactic acid-derived antimicrobial activity from that of other active components in the CFS.

To get further insights into the protein content of our CFS and their possible contribution to the overall antibacterial activity we performed a label-free proteomic analysis of unfractionated CFS.

Among the identified proteins, cell wall hydrolases are of particular interest. These enzymes are typically membrane-bound and play various roles in the bacterial life cycle, including cell wall biosynthesis, cell separation, cell adhesion, and virulence (Chapot-Chartier and Kulakauskas, 2014; Vermassen et al., 2019). Notably, they have the ability to degrade the bacterial cell wall, causing bacteriolysis and ultimately leading to cell death (Parisien et al., 2008). Phage lysins have been extensively studied, as they are fundamental to bacteriophage function (O’Flaherty et al., 2009; Vázquez et al., 2018). Although these enzymes have shown potential mostly against Gram-positive bacteria, recent strategies have made lysins effective against Gram-negative bacteria as well (Vázquez et al., 2018). However, bacterial lysins have not been as thoroughly investigated as phage lysins and data that support their specific role remain limited. Interestingly, previous studies suggest that the immune system may exploit this mechanism as well; cationic agents released by neutrophils have been hypothesized to activate bacterial hydrolases, thereby triggering autolysis and contributing to bacterial clearance (Ginsburg, 2001).

The ability to switch to the biofilm mode of growth is a major factor in *P. aeruginosa*’s ability to cause persistent infections and resist antibiotic treatment, posing significant challenges in clinical settings (Høiby et al., 2024). The extracellular matrix of the biofilm limits the penetration of antibiotics, while the bacteria within biofilms can exhibit a slower growth rate, reducing the effectiveness of antibiotics that target rapidly dividing cells. Antibiotic tolerance of biofilms can also be caused by the formation of the so-called persister-cells, a subpopulation of slowly or non-dividing bacteria that are virtually insensitive to all antibiotics, but preserve the ability to reacquire a vegetative state when antibiotic treatment is terminated (Ciofu and Tolker-Nielsen, 2019). Finding novel strategies against *P. aeruginosa* biofilms is therefore of paramount importance to overcome biofilm resilience to treatment and improve healthcare. In the present study, we investigated the ability of CFS fractions to target preformed biofilms of *P. aeruginosa* as compared to unfractionated CFS. Similarly to what was observed with planktonic bacteria, the CFS ≤ 3kDa fraction was the one mainly responsible for the biofilm biomass reduction evaluated as crystal violet staining after 24 h of treatment. Previous work has demonstrated that lactic acid exerts an antibiofilm effect against *Escherichia coli* and other food-borne pathogens’ biofilms, suggesting that such component of the CFS ≤ 3kDa fraction might be responsible, at least in part, for the antibiofilm activity of CFS against our *P. aeruginosa* strain. Nevertheless, differently to what was observed with planktonic bacteria, even the CFS > 3kDa fraction caused a statistically significant reduction of biofilm biomass when used at a dilution of 1:4 suggesting that components other than lactic acid may contribute to the antibiofilm activity of unfractionated CFS.

Among the proteins identified in the CFS in this study, an L,D-transpeptidase and an Ala-D-Ala carboxypeptidase could potentially contribute to the antibiofilm activity observed in the >3 kDa fraction of the CFS (Marques et al., 2024). In silico analysis of *L. rhamnosus* GG and *Lactobacillus acidophilus* NCFM identified a serine-type D-Ala-D-Ala carboxypeptidase and an L,D-transpeptidase with high affinity for AgrB and AgrC proteins from *Listeria monocytogenes*. AgrB and AgrC are well-known components of the Accessory Gene Regulator (Agr) system, which regulates quorum sensing and biofilm formation. The Agr system has been extensively studied in *Staphylococcus aureus*, where its inhibition has been shown to reduce virulence and biofilm formation (Tan et al., 2018). Given its probable presence in *Clostridium perfringens* and *Enterococcus faecalis*, targeting this pathway could have broader applications beyond *S. aureus*, potentially serving as a strategy against multiple bacterial species (Gray et al., 2013).

The standard CV assay is indicative of the total biofilm biomass, but it does not give any information on whether the attached cells are viable or non-viable. Therefore, we further investigated the anti-biofilm activity of unfractionated and fractionated CFS via confocal microscopy following live/dead staining. The results obtained paralleled those obtained with CV staining, indicating a direct killing effect as a possible mechanism of CFS action towards biofilm-embedded bacterial cells. We have previously reported that *L. rhamnosus* CFS display killing activity against *P. aeruginosa* persister-cells isolated from mature biofilms (Bianchi et al., 2024) and reduces the production of QS-regulated virulence factors by *P. aeruginosa* strains isolated from CF patients (Pompilio et al., 2024). Thus, the antibiofilm effect of CFS and their fractions reported herein could also rely on their ability to kill persister-cells in the biofilm and/or to interfere with QS-signals involved in biofilm formation.

Another interesting aspect addressed in this study was the immunomodulatory properties of unfractionated and fractionated CFS. Intense and uncontrolled inflammation is considered a feature of *P. aeruginosa* infections (Lin and Kazmierczak, 2017). Although inflammation is necessary for bacterial clearance, an exuberant inflammatory response, with the release of elevated levels of pro-inflammatory cytokines, leads to immune-mediated destruction of lung parenchyma in diseases like cystic fibrosis or chronic obstructive pulmonary disease (COPD) (Lin and Kazmierczak, 2017).

Reducing bacterial load meanwhile modulating inflammation seems therefore necessary to control *P. aeruginosa* infections. In this study, we showed a strong ability of unfractionated CFS to dampen the release of two major pro-inflammatory cytokines (IL-6 and TNF-α) by human PBMC stimulated with *P. aeruginosa* LPS. Interestingly, such ability was entirely ascribed to the CFS ≤ 3kDa fraction, the one with the highest content of lactic acid. These results are in agreement with a recent study reporting lactate as a metabolic mediator able to influence immune cell function (Caslin et al., 2021). Specifically, lactate and the associated H^+^ ions, which are the predominant forms at physiological pH, have been reported to suppress glycolytic ATP production in immune cells, contributing to reduced inflammatory cell signalling and mediator secretion (Caslin et al., 2021). In monocytes and macrophages, lactic acid and acidification can also suppress specific receptor signalling cascades inhibiting lipopolysaccharide (LPS)-induced cytokines and chemokines and toll-like receptor (TLR)-4 mediated inflammasome assembly (Dietl et al., 2010).

Notably, the immunomodulatory effects of lactobacilli-derived products have been further demonstrated in different models of inflammation-related diseases. A recent study on a *Lactobacillus*-based inhaled biotherapeutic product highlighted its ability to mitigate neutrophilic inflammation in murine models of bronchopulmonary dysplasia and COPD, significantly reducing pro-inflammatory markers and improving lung function (Nicola et al., 2024). These findings reinforce the concept that lactobacilli-derived metabolites, including lactic acid, can modulate inflammatory responses across different disease settings.

In contrast to the CFS ≤ 3kDa fraction, the CFS > 3kDa showed no ability to reduce IL-6, TNF-α and IL-10 release by LPS-stimulated PBMC but, rather, induced itself high levels of such cytokines that summed up to those induced by LPS. These findings suggest the presence in the CFS > 3kDa fraction of high molecular weight components and/or cell wall fragments able to directly interact with host cell receptors (e.g. TLR-2, TLR-4) leading to a stimulatory response which is masked by ≤ 3kDa components in unfractionated CFS.

Some of the proteins identified in the supernatants could also contribute to the observed cytokine response. For instance, proteomic analysis revealed the presence of ABC transporter substrate-binding proteins (**Supplementary Material**). Interestingly, Kurokawa et al. demonstrated that a substrate-binding ABC transporter of *S. aureus* (the triacylated SitC lipoprotein) is capable of stimulating TLR2, triggering the production of TNF-α and IL-6 in mouse peritoneal macrophages (Kurokawa et al., 2009). Additionally, they isolated two other native ABC transporter substrate-binding lipoproteins from *Bacillus subtilis* and *Micrococcus luteus*, both of which were found to activate TLR2. Secreted factors of different lactic acid bacteria were also found to induce cytokine (IL-10 and TNF-α) production by THP1-differentiated human macrophages in a species and strain-specific manner (Ren et al., 2020). More specifically, immune-regulating properties of bacterial CFS were found to be highly dependent on their activating abilities of TLR2 signalling. TLR2-signaling activities of CFS from most bacterial species were weakened by proteinase and/or heat treatment, suggesting that at least a portion of TLR2 ligands in these bacterial CFS samples were proteins (Ren et al., 2020).

We have previously reported that *P. aeruginosa* biofilms stimulate the production of both pro-inflammatory and anti-inflammatory cytokines by human PBMC at higher levels than their planktonic counterparts do (Kaya et al., 2021). Similar findings were reported by Ciornei and coworkers, who demonstrated that the acquisition of the biofilm mode of growth by *P. aeruginosa* is accompanied by structural modification of its LPS that renders it particularly pro-inflammatory (Ciornei et al., 2010). Therefore, we aimed to test the immunomodulatory properties of CFS and their fractions using intact *P. aeruginosa* cells derived from mature biofilms as a stimulus for PBMC. Biofilm-derived *P. aeruginosa* induced higher levels of pro-inflammatory cytokines as compared to LPS alone, yet CFS and CFS ≤ 3kDa fraction strongly abrogated such pro-inflammatory response, confirming their immunomodulatory potential also towards complete bacterial cells.

To exclude the possibility that the observed inhibition of cytokine secretion by the CFS ≤ 3kDa fraction was due to cell death, PBMC viability was tested in the presence of unfractionated and fractionated CFS, with or without the addition of the pro-inflammatory stimuli (LPS or biofilm-derived *P. aeruginosa*). All the combinations, except unfractionated CFS alone, at the dilution of 1:8 (approximately 15 mM lactic acid), had no effect on cell viability evaluated as PI incorporation. These findings are in agreement with a previous study that reported no cytotoxicity of lactic acid up to 20 mM towards human macrophages (Dietl et al., 2010).

In conclusion, this study suggests that *L. rhamnosus*-derived postbiotics, in the form of cell-free supernatants, have promising antimicrobial potential against *P. aeruginosa*, as well as a noteworthy dual role in anti-inflammatory activity. In summary, the antibacterial, antibiofilm, and immunomodulatory activities observed were primarily linked to the ≤ 3 kDa fraction, which is rich in lactic acid. However, proteomic analysis identified high-molecular-weight components with potential immunomodulatory and antibiofilm properties, further emphasizing the multifaceted effects of CFS and the need for further research to elucidate the synergistic interactions among its components. Ongoing studies will specifically investigate the ≥ 3 kDa fraction by separation and purification methodologies to identify biomolecules of interest with anti-infective and/or immunomodulatory potential (e.g. bacteriocins, exopolysaccharides, cell wall fragments, etc) and to proceed to their biochemical and functional characterization.

## Data Availability

The raw data supporting the conclusions of this article will be made available by the authors, without undue reservation.
