# Supplementary material for: Biotherapeutic potential of different fractions of cell-free supernatants from Lacticaseibacillus rhamnosus against Pseudomonas aeruginosa
Source: Front Cell Infect Microbiol. 2025 Jun 30;15:1608897. doi: 10.3389/fcimb.2025.1608897 (PMC12256502; doi:10.3389/fcimb.2025.1608897)
Supplement: Supplementary file 1 [file DataSheet1.pdf]

Supplementary Table 1: The complete list of the 37 high-confidence proteins identified from the cell free supernatant (CFS) using label-free qualitative proteomic analysis.

Gene Names	Protein IDs (UniProt)	Description	Function
NIM79_008335	A0AAP7FZ74	Oligopeptide ABC transporter substrate-binding protein	Molecular Transport
AWJ15_04035	A0A180BN67	Cell division protein FtsI (Penicillin-binding protein 2)	Cell Wall Structure and Synthesis
HWN39_03905	A0A5P5ZAV2	ABC transporter substrate-binding protein	Molecular Transport
NIM79_009755	A0AAP7FXK3	Peptide ABC transporter substrate-binding protein	Molecular Transport
AWJ15_11830	A0A5P5Z8J3	Hydrolase	Enzymes and Catalytic Activity
AWJ15_06895	A0A0E3CLJ0	FMN-binding domain-containing protein	Enzymes and Catalytic Activity
H0N82_09730	A0A853J5K3	Peptide ABC transporter substrate-binding protein	Molecular Transport
AWJ15_06515	A0A179XE40	Hydrolase	Enzymes and Catalytic Activity
msp1	A0A5P5Z8J8	Cell wall hydrolase P75	Enzymes and Catalytic Activity
NIM79_013785	A0AAP7KLK2	FliK family flagellar hook-length control protein	Flagellar assembly proteins
NIM79_013770	A0AAP7FZN1	Uncharacterized protein	Uncharacterized protein
wzr	C1J9J6	LCP family protein	Cell Wall Structure and Synthesis
H0N82_00730	A0A171J5F2	Serine protease	Enzymes and Catalytic Activity
ugpB_3	A0A6N3CD19	ABC transporter substrate-binding protein	Molecular Transport
NIM79_010085	A0AAP7FXE8	NlpC/P60 family protein	Enzymes and Catalytic Activity
NIM79_005620	A0AAP7FZQ4	DUF3862 domain-containing protein	Uncharacterized protein
AWJ15_08000	A0A180A7K3	L,D-transpeptidase family protein	Cell Wall Structure and Synthesis
AWJ15_11650	A0A5P5Z942	Serine-type D-Ala-D-Ala carboxypeptidase	Cell Wall Structure and Synthesis
AWJ15_13520	A0A807RTX2	ABC transporter substrate-binding protein	Molecular Transport
NIM79_000565	A0AAP7FZ91	Cell wall hydrolase P40	Enzymes and Catalytic Activity
prsA	A0A0D6UDC7	Foldase protein PrsA	Chaperone
AWJ15_13045	A0AAC9Q2F8	Extracellular protein	Extracellular protein
NIM79_000015	A0AAP7FVW8	Amino acid ABC transporter substrate-binding protein/permease	Molecular Transport
NIM79_011125	A0AAP7FZ90	LCP family protein	Cell Wall Structure and Synthesis
NIM79_000745	A0AAP7G0S9	MMPL family transporter	Molecular Transport
NIM79_006260	A0AAP7KKM8	Peptide ABC transporter substrate-binding protein	Molecular Transport
NIM79_012790	A0AAP7KKW8	DUF1002 domain-containing protein	Uncharacterized protein
AWJ15_02715	A0A2A5L9L1	DNA-binding protein HU	DNA-binding proteins
rplL	A0A2A5L7S3	Large ribosomal subunit protein bL12	Ribosomial protein
AWJ15_08830	A0A0D6U787	LemA family protein	Transmembrane protein
AWJ15_11820	A0A0D6UAI2	LCP family protein (LytR family transcriptional regulator)	Cell Wall Structure and Synthesis
CYJ91_00010	A0A0E3CMF0	Uncharacterized protein	Uncharacterized protein
AWJ15_03205	A0A5P5ZC93	DUF5590 domain-containing protein	Uncharacterized protein
AWJ15_09180	A0A807RTK9	YxeA family protein	DNA-binding proteins
NIM79_008535	A0AAP7FY22	Uncharacterized protein	Uncharacterized protein
NIM79_007510	A0AAP7FZZ8	Penicillin-binding protein	Cell Wall Structure and Synthesis
NIM79_007915	A0AAQ2N1I8	N-acetylmuramoyl-L-alanine amidase	Enzymes and Catalytic Activity
